# A UK wide cohort study describing management and outcomes for infants with surgical Necrotising Enterocolitis

**DOI:** 10.1038/srep41149

**Published:** 2017-01-27

**Authors:** Benjamin Allin, Anna-May Long, Amit Gupta, Marian Knight, Kokila Lakhoo, British Association of Paediatric Surgeons Congenital Anomalies Surveillance System Necrotising Enterocolitis Collaboration, Marcin Kazmierski, Simon Kenny, Joana Lopes, Eleri Cusick, Gilian Parsons, Amanda McCabe, Manasvi Upadhyaya, Gregor Walker, Paulo De Coppi, Sania Besarovic, Hemanshoo Thakkar, Lucinda Tullie, Jonathan Sutcliffe, Bala Eradi, Andrew Ross, Nomsa Maphango, Sandeep Motiwale, Adnan Salloum, Caroline Pardy, Ramy Waly, Paul Charlesworth, Ross Craigie, Anupam Lall, Richard Lindley, Navroop Johal, Ike Njere, Alan Mortell, Bip Nandi, Abigail Jones, Dina Fouad, Yew-Wei tan, Dorothy Kufeji, Joanna Stanwell, Bhanu Lakshminarayanan, David Burge, Charlotte Wetherill, Anindya Niyogi, Chris Parsons, Miriam Doyle, Alex Turner, Ian Yardley, Ram Shrestha, Dhanya Mullassery, Saravankumar Paramalingham, Simone Ragazzi

**Affiliations:** 1 National Perinatal Epidemiology Unit, Old Road Campus, Headington, Oxford, OX37LF, UK; 2 Department of Paediatric Surgery, Oxford Children’s Hospital, Headley Way, Oxford, OX39DU, UK; 3 Neonatal Intensive Care Unit, Oxford Children’s Hospital, Headley Way, Oxford, OX39DU, UK.; 4 Addenbrooke’s Hospital, Cambridge, United Kingdom; 5 Alder Hey Children’s Hospital, Liverpool, United Kingdom; 6 Birmingham Children’s Hospital, Birmingham, United Kingdom; 7 Bristol Royal Hospital for Children, Bristol, United Kingdom; 8 Chelsea and Westminster Hospital, London, United Kingdom; 9 Edinburgh Royal Hospital for Sick Children, Edinburgh, United Kingdom; 10 Evelina Children’s Hospital. London, United Kingdom; 11 Glasgow Royal Hospital for Sick Children, Glasgow, United Kingdom; 12 Great Ormond Street Hospital for Sick Children, London, United Kingdom; 13 Hull Royal Infirmary, Hull, United Kingdom; 14 King’s College Hospital, London, United Kingdom; 15 Leeds General Infirmary, Leeds, United Kingdom; 16 Leicester Royal Infirmary, Leicester, United Kingdom; 17 Norfolk and Norwich University Hospital, Norwich, United Kingdom; 18 Our Lady’s Hospital for Sick Children, Crumlin, Ireland; 19 Queen’s Medical Centre, Nottingham, United Kingdom; 20 Royal Aberdeen Children’s Hospital, Aberdeen, United Kingdom; 21 Royal Alexandra Children’s Hospital, Brighton, United Kingdom; 22 Belfast Hospital for Sick Children, Belfast, United Kingdom; 23 Royal London Hospital, London, United Kingdom; 24 Royal Manchester Children’s Hospital, Manchester, United Kingdom; 25 Royal Victoria Infirmary, Newcastle, United Kingdom; 26 Sheffield Children’s Hospital, Sheffield, United Kingdom; 27 Southampton Children’s Hospital, Southampton, United Kingdom; 28 St George’s Hospital, London, United Kingdom; 29 The Children’s University Hospital, Dublin Ireland; 30 University Hospital of Wales, Cardiff, United Kingdom

## Abstract

The Royal College of Surgeons have proposed using outcomes from necrotising enterocolitis (NEC) surgery for revalidation of neonatal surgeons. The aim of this study was therefore to calculate the number of infants in the UK/Ireland with surgical NEC and describe outcomes that could be used for national benchmarking and counselling of parents. A prospective nationwide cohort study of every infant requiring surgical intervention for NEC in the UK was conducted between 01/03/13 and 28/02/14. Primary outcome was mortality at 28-days. Secondary outcomes included discharge, post-operative complication, and TPN requirement. 236 infants were included, 43(18%) of whom died, and eight(3%) of whom were discharged prior to 28-days post decision to intervene surgically. Sixty infants who underwent laparotomy (27%) experienced a complication, and 67(35%) of those who were alive at 28 days were parenteral nutrition free. Following multi-variable modelling, presence of a non-cardiac congenital anomaly (aOR 5.17, 95% CI 1.9–14.1), abdominal wall erythema or discolouration at presentation (aOR 2.51, 95% CI 1.23–5.1), diagnosis of single intestinal perforation at laparotomy (aOR 3.1 95% CI 1.05–9.3), and necessity to perform a clip and drop procedure (aOR 30, 95% CI 3.9–237) were associated with increased 28-day mortality. These results can be used for national benchmarking and counselling of parents.

Necrotising enterocolitis (NEC) predominantly affects low birth weight, premature infants^1^, and is a significant cause of major morbidity and mortality in developed countries^1^,^2^. In America, the Vermont Oxford Network (VON) has made significant inroads into ensuring that outcomes for infants with NEC are accurately reported. However, whilst their work provides a robust overview, they do not report nuanced data relating to intra-operative findings or management strategies, making it more difficult to fully assess the burden of disease associated with surgical NEC. Differences in surgical culture between developed countries may also make it difficult to automatically apply their results to other Western populations. In 2011, when the National Confidential Enquiry into Patient Outcomes and Death (NCEPOD) report identified that *“difficulty in decision making during both medical management and the decision to operate in babies with NEC”*^3^ was a key factor in limiting outcomes for infants with NEC in the UK, there existed no national data collection system in the UK. Without such data, it is impossible to robustly benchmark outcomes for infants with surgical NEC in the UK and Ireland, or for the Royal College of Surgeons to meaningfully pursue their aim of using outcomes from infants with surgical NEC as a contributor to revalidation of neonatal surgeons. It is also exceptionally difficult to accurately counsel parents of infants with NEC.

There is significant on-going debate as to whether NEC and spontaneous intestinal perforation (SIP) should be considered as one or two separate conditions. Certainly, evidence from the VON, would suggest that whilst there are statistically significant differences in risk factors for development of the two conditions, the clinical difference in these factors would make it almost impossible to reliably differentiate the two pre-operatively^4^. Mortality for infants with SIP does however appear to be lower than that of infants with NEC^4^. For the purposes of determining the need for surgical intervention in these infants, it therefore appears prudent to treat them as one condition, whilst for the purposes of post-operative prognostication, treating them separately would appear more appropriate.

The aims of this prospective nationwide cohort study, therefore, were to document the number of infants in the United Kingdom and Ireland who underwent or were judged to need surgical intervention for NEC or SIP, describe in detail their surgical management and outcomes to 28 days post decision to intervene, and assess the association of key pre-operative and operative factors with 28 day mortality.

## Methods

### Ethical Approval

Ethical approval for the study was granted by the National Research Ethics Service Committee South Central – Oxford A (Study reference number 12/SC/0416). As this was an observational study no experimentation or intervention took place, therefore approval of experimental protocols by institutional or licensing committees was not required. All data collected was anonymous, and in accordance with the decision of the Research Ethics Service Committee, no consent for participation was required from study participants.

### Study Design and setting

A nationwide, multi-centre, prospective cohort study was conducted between the 1^st^ of March 2013 and the 28^th^ of February 2014 utilising the British Association of Paediatric Surgeons Congenital Anomalies Surveillance System (BAPS-CASS) to identify infants undergoing, or judged to need, surgical management of NEC or SIP in all 27 paediatric surgical centres in the United Kingdom and Ireland.

### Participants

Cases were identified via monthly reporting cards sent to lead clinicians at participating centres, with detailed data collection forms completed in response to notification of a case. Lead clinicians were asked to return a ‘null’ card specifying if there had been no cases in their institution that month. If cards were not returned for three months, we contacted lead surgeons by telephone or email to obtain the missing case reports. All data were anonymous, and were double entered into a customised database. Duplicates were excluded by comparing reporting hospital, mother’s year of birth, and date of first operation. If any data items were missing, or fell outside pre-specified ranges, we contacted clinicians to obtain the required information. This data collection methodology is described in further detail in previous publications^5^.

Infants were eligible for inclusion in the study if they were deemed to require surgical intervention for NEC, regardless of whether that intervention was actually delivered. NEC was diagnosed in one of three ways:

1. On visual inspection of the bowel at time of laparotomy
2. At the time of post-mortem
3. Clinically using the Vermont-Oxford Criteria (at least one of bilious gastric aspirate or emesis, abdominal distension, or occult or frank rectal bleeding, combined with at least one radiological finding of either pneumatosis intestinalis, hepatobiliary gas, or pneumoperitoneum^6^).

Infants who were diagnosed with NEC clinically, but who at the time of surgery were determined to have a cause for their symptoms other than NEC or SIP were excluded from the analysis. This case definition was used in order to allow identification of a sub-set of infants with NEC who have a severe form of the disease, and in whom the condition is robustly definable. By contrast, due to the non-specific presenting clinical features, it is very difficult to identify or define infants with ‘medically managed NEC’.

The case definition used deliberately encompasses infants with both NEC and SIP, as it is not possible to reliably differentiate the two prior to laparotomy. For the purposes of investigating associations between presenting features and 28-day mortality, these infants were assessed as one group. However, as there is evidence from the VON that outcomes are different for infants with NEC, sub-group analyses were performed when describing primary and secondary outcomes.

It is possible that the case definition used may also capture infants with other conditions such as volvulus. Where these conditions were identified at laparotomy, we have excluded them from the analysis.

### Outcomes and data sources

Our primary outcome of interest was mortality prior to twenty-eight days post initial decision to intervene surgically, with secondary outcomes including development of a Clavien-Dindo grade two^7^ or above complication prior to 28 days, (i.e. those complications that require pharmacological intervention, surgical intervention, are life threatening, or cause death), parenteral nutrition use at twenty-eight days, and discharge home prior to twenty-eight days. Sub-group analyses were performed for infants with NEC confirmed at laparotomy, and infants with SIP confirmed at laparotomy. In order to allow direct comparison with infants assessed as part of the Vermont-Oxford Network studies, mortality prior to 28 days post decision to intervene surgically was also calculated in the sub-group of infants with a birth-weight of 401 g to 1500 g.

All data were collected by reporting clinicians using appropriate medical records, including patent notes, imaging and laboratory data.

### Statistical Analysis

Rates for each of the primary and secondary outcome measures were calculated based upon the number of infants with information returned for that variable.

Odds ratios and 95% confidence intervals were calculated for independent variables considered likely to have an impact on mortality at twenty-eight days on the basis of previous studies or clinical hypothesis. Univariable logistic regression analysis was used initially. Pre-operative factors investigated were; gender, ethnicity, umbilical artery reversed end diastolic flow, umbilical artery absent end diastolic flow, antenatal corticosteroid use, caesarean delivery, multiple foetuses, gestational age at birth (completed weeks), birth weight, radiological evidence of perforation, abdominal wall erythema or discolouration, inotrope use at presentation, need for ventilation at presentation, antacid use at presentation, cardiac surgery other than that for a patent ductus arteriosus (PDA), non-cardiac congenital anomaly, transfer in to surgical centre, PDA ligation performed, Indomethacin administration for PDA closure, umbilical catheter ever used, enteral feeding at time of diagnosis, formula milk use at diagnosis, and blood transfusion less than 2 weeks prior to diagnosis. Operative factors investigated were: spontaneous intestinal perforation diagnosed at time of operation, necessity to resect the ileocaecal valve, necessity for intra-abdominal drain placement prior to laparotomy, and procedure performed at laparotomy.

Two multi-variable logistic regression analysis models were developed. The first was used to control for the potential confounding effect of pre-operative independent variables on mortality at 28 days post intervention. Factors that had been shown on univariable analysis to significantly impact on mortality at 28-days post intervention, as defined by a p-value of ≤ 0.2 were added into the model in a forward stepwise manner in order of statistical significance. However, because of the weight of pre-existing evidence of their impact on mortality, an a priori decision was made that gestational age would be included in all models, and birth weight would be investigated for inclusion in the regression analysis model regardless of whether it met the statistical criteria. No evidence of departure from linearity was identified for gestational age or birth-weight, and therefore they were analysed as continuous variables whilst developing the multivariable model. Independent variables were dropped from the model if they did not statistically significantly affect its fit, as defined by a p-value of > 0.1 on likelihood ratio testing.

The second model was developed through the addition of operative variables to the pre-operative model, with the intention of investigating the impact these variables had on mortality on 28 days. Operative independent variables were assessed for inclusion in the model using the same criteria as for the pre-operative model. Because of the existing evidence from the Vermont Oxford Network that infants with NEC and SIP have different mortality rates, an a priori decision was made that diagnosis of SIP would be included in all models.

Complete case analysis was used for development of both models. All statistical analysis was performed using Stata version 13 (StataCorp. 2009. Stata: Release 13. Statistical Software. College Station, TX: StataCorp LP).

## Results

### Participants

In the defined reporting period, 236 infants were identified as eligible for inclusion in the study. In the same period, there were 847100 live born infants in the UK and Ireland^8^,^9^,^10^,^11^. This therefore represents an incidence of surgically managed NEC of 27.9 per 100,000 live births. The characteristics of the infants are shown in Table 1. Twenty-six infants (11%) had a non-cardiac congenital anomaly. These included amongst others, renal tract anomalies, limb anomalies, neurological anomalies or cranial malformations, gastrointestinal anomalies, facial anomalies, situs inversus, genetic and chromosomal anomalies, and tracheomalacia.

### Operative findings and management

Overall, two hundred and twenty four infants (95%) underwent an emergency laparotomy. Thirty-two of these infants (14%) were identified as having a single intestinal perforation within otherwise healthy bowel and were therefore determined by the operating surgeon to have SIP. 189 infants (84%) had operative findings in keeping with NEC. There were three infants (1%) where it was unclear at the time of operation whether the infant had NEC or SIP.

**Figure 1:**
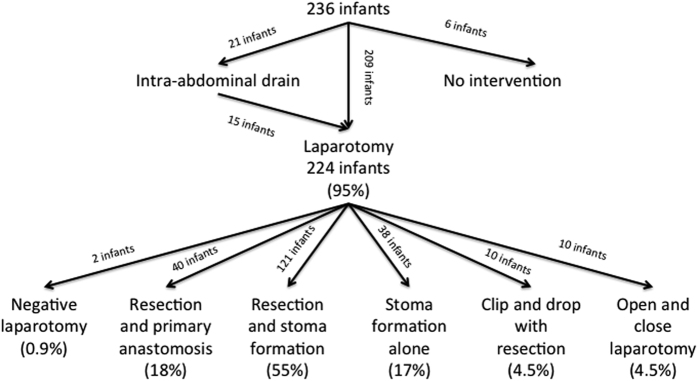
Management of infants requiring surgical intervention for necrotising enterocolitis.

121 of the infants who underwent laparotomy (54%) required intestinal resection and stoma formation, making this the most commonly performed procedure (Table 2 and Fig. 1). Conversely, primary peritoneal drainage was only used in 21 infants (9%), 15 of whom (71%) subsequently proceeded to laparotomy. There were a further 6 infants (3%) who underwent no surgical intervention despite it initially being deemed necessary. Three of these infants (50%) were deemed too sick for surgical intervention, and died prior to it being undertaken, whilst the remaining three (50%) recovered sufficiently prior to surgical intervention for it to be deemed no longer necessary. These three infants survived. This gives a total of 12 infants (5%) who did not undergo a laparotomy, and in whom it was therefore not possible to determine whether they had SIP or NEC. There were however no statistically significant differences in procedure undertaken when infants with laparotomy confirmed SIP were compared to those with laparotomy confirmed NEC (Table 2).

### Mortality, post-operative complications, parenteral nutrition use, and discharge home

Within the cohort as a whole, 43 infants (18%) died prior to 28 days post decision to intervene surgically (Table 3). The median time between decision to intervene and death was 2 days (IQR 0–10 days). Cause of death as recorded on the death certificate was reported as being due to NEC in thirty-one infants (72%), sepsis (not recorded as secondary to NEC) in four infants (9%), other causes related to extreme prematurity in six infants (14%), and unknown causes in two infants (5%). Of the 188 infants with a birth-weight of between 401 g and 1500 g, and who would therefore be eligible for inclusion in the Vermont Oxford studies, 34 died prior to 28 days, also giving a mortality rate of 18%.

Of the 224 infants who underwent a laparotomy, nine (4%) experienced bleeding intra-operatively from a liver injury. Sixty infants (27%) experienced a total of 74 post-operative complications of Clavien-Dindo grade II or above. Seven of these infants (12%) experienced two complications, two infants (3%) experienced three complications, and one infant (2%) experienced four complications. Stoma complications were the most common, affecting 27 infants (12%), followed by wound complications (20 infants, 8%), and intra-abdominal sepsis (12 infants, 5%). Of the 193 infants who were alive at 28 days, 67 (35%) were confirmed to no longer require parenteral nutrition, and eight infants from the cohort as a whole (3%) had been discharged home.

### Pre-operative factors associated with increased twenty-eight day mortality

**Figure 2:**
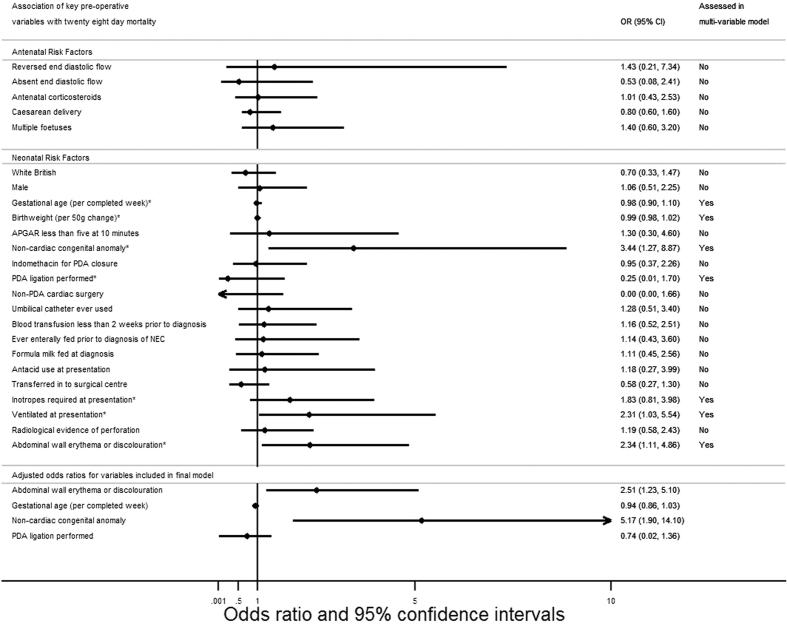
Association of pre-operative characteristics with 28-day mortality.

Results of the univariable analysis are shown in Supplementary Table S1. Following adjustment, abdominal wall erythema or discolouration (adjusted odds ratio 2.51, 95% CI 1.23–5.1, p = 0.011), and presence of a non-cardiac congenital anomaly (adjusted odds ratio 5.17, 95% CI 1.9–14.1, p = 0.001) were statistically significantly associated with an increased odds of mortality at 28 days post-surgical intervention (Fig. 2 and Table 4). Complete case analysis was undertaken based upon 234 infants (99%) with complete data.

### Association of operative findings and interventions with twenty-eight day mortality

**Figure 3:**
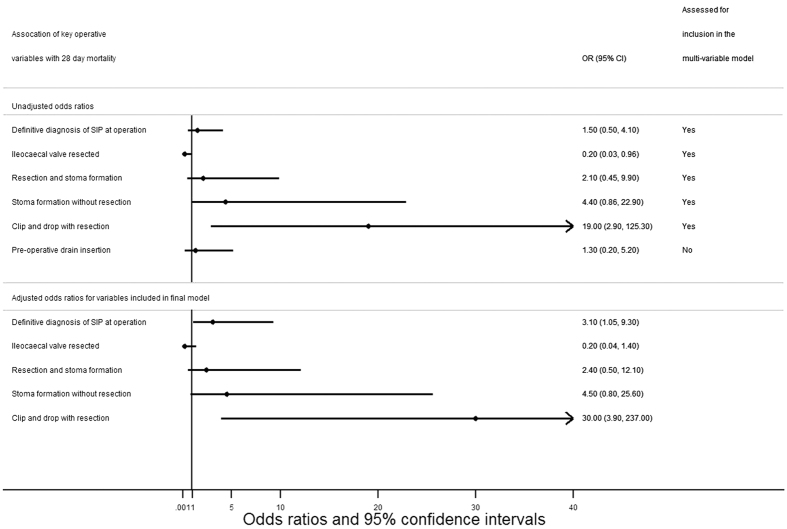
Association of key operative findings and management strategies with 28-day mortality.

Following adjustment for resection of the ileocaecal valve, choice of definitive surgical procedure, the four variables forming the pre-operative model, and the diagnosis of SIP at the time of operation, two operative factors were identified as being associated with an increased risk of 28-day mortality. Operative diagnosis of SIP was associated with a statistically significantly increased odds of 28-day mortality (adjusted OR 3.1, 95% CI 1.05–9.3, p = 0.04), as was performance of clip and drop procedure with intestinal resection, when compared to the operation that was associated with the lowest rate of 28-day mortality, resection and primary anastomosis (adjusted OR 30.2, 95% CI 3.9–237, p = 0.001) (Fig. 3 and Table 5). Complete case analysis was used for development of the final model, and therefore data from 17 of the 224 infants who underwent a laparotomy (7.5%) were not used in its development.

## Discussion

The incidence of surgical NEC in the UK as estimated by this study is 27.9 cases per 100,000 live births. As the overall incidence of NEC is approximately 109 per 100,000 live births^12^, this would suggest that around one in four infants with NEC require surgical intervention. Given the previous validation of the BAPS-CASS data collection methodology^13^, and the fact that this figure is in line with previously reported studies^14^,^15^,^16^, we believe these incidences to be an accurate reflection of the incidence of surgical NEC in the UK. The most common surgical intervention for these infants was stoma formation and intestinal resection, and in contrast to the VON studies, primary peritoneal drainage was rarely used, and in the few instances where it was, was most commonly a resuscitative as opposed to definitive procedure^17^. This pattern held true for infants with SIP, as well as for infants with NEC, suggesting that there may be a shift away from the traditional doctrine that infants with SIP undergo a primary anastomosis. Overall, almost one in five infants with surgical NEC died prior to 28 days, and one in four developed a significant complication. However, one in three attained full enteral independence, and 3% were discharged home prior to 28 days. Reinforcing the findings of Dyke *et al*., this study suggests that infants with non-cardiac congenital anomalies are at a higher risk of early mortality^18^. The presence of abdominal wall erythema or discolouration at presentation was also associated with increased 28-day mortality.

Due to the precision of the case definition used, and the detailed data collected, this study has allowed for a robust description of the management and early outcomes of infants with surgical NEC. Whilst the factors identified as being associated with increased 28-day mortality are useful for informing clinical decision-making and counselling of parents, the amount of variation in mortality (r^2^) that is explained by the model is relatively low, at 19%. This suggests there are other important risk factors that we have been unable to quantify. Such factors may include variables such as pH and thromboyctopaenia, which we have not ben able to collect data on. Although our study represents every infant reported as having been treated for surgical NEC in the UK and Ireland between 1^st^ of March 2013,and 28^th^ of February 2014, there are still only 236 cases. It is possible therefore that we were unable to detect some associations with mortality as statistically significant due to a lack of study power. As with other observational studies of surgical interventions, another limitation of this work, is that inclusion in the study is defined by the threshold at which different clinicians deem surgical intervention appropriate. This potentially leads to differences in case mix between participating centres, and also between this study and others of surgical NEC. The threshold for operating is likely to vary based upon culture in an institution or country, and therefore outcomes between studies may vary due to the culture in which the study was conducted. This however is a limitation of all studies of surgical NEC, and not one that can be addressed until we have developed sufficient evidence from observational studies to conduct a randomised controlled trial of different surgical interventions.

Whilst giving some optimism when compared to previously published work^1^,^3^,^19^,^20^,^21^,^22^,^23^,^24^, these results would suggest there is still some way to go in improving outcomes for infants with NEC. Further improving these outcomes is reliant on highlighting infants who are at higher risk of adverse events. When choice of operative procedure is added to the multi-variable model, the amount of variation in mortality explained by the model rises from 9 to 19%. This suggests that the operative procedure performed, or factors influencing a surgeon’s choice of procedure, account for approximately 10% of variation in 28-day mortality. We believe that the latter of these will account for the majority of that variation. This would therefore suggest that there are factors which influence a surgeon’s choice of operation, and which are associated with changes in 28-day mortality, but which we have been unable to control for. If, as per the NCEPOD report recommendations^3^, mortality for infants with NEC is to be improved through identification of the optimal point for surgical intervention, further definition of these unknown confounding factors must be undertaken.

Comparison of our work with that of the Vermont-Oxford Network (VON) reveals several key differences. The overall mortality rate of 18% seen in our study was lower than the 35% seen in the VON study^17^. Some of this difference will be explained by the fact that we only report outcomes up to 28 days post decision to intervene surgically, whereas the VON reports to one year. Even accounting for this, an absolute difference in mortality of 17% between the cohorts appears larger than expected, but could be explained by three factors. Firstly, that there is a different threshold for intervening surgically between the UK and the US, and that very sick infants with NEC in the US are undergoing surgical intervention, whereas in the UK they are being deemed too sick for surgical intervention, and therefore not meeting the criteria for inclusion in studies of surgical NEC. Secondly, whilst a Cochrane systematic review consisting of approximately 185 patients has suggested that primary peritoneal drainage (PPD) as an initial intervention is not associated with a different mortality rate to laparotomy as an initial intervention, both our work, and previous work by Hull *et al*. have shown that use of primary peritoneal drainage (PPD) as a definitive procedure, without progression to laparotomy, is associated with a significantly higher rate of mortality than laparotomy^17^. PPD alone is generally used when infants are deemed too sick to undergo laparotomy, and in the VON studies, was used in approximately 17% of infants with surgical NEC^17^, whereas in this study, it was used in approximately 3% of infants. This differential rate of PPD as a definitive procedure could imply that there is a sicker population of infants with surgical NEC in the VON studies, that American surgeons have a higher threshold for undertaking a laparotomy, or that infants who in the US are receiving PPD are deemed too sick in the UK to undergo any surgical intervention at all. Either way, the differential rate of PPD as definitive treatment may account for a significant proportion of the differences in mortality rate demonstrated. Finally, there may be a genuine difference in mortality rate between infants with surgical NEC in the UK and in the US, and this may be accounted for by differences in choice of surgical intervention, antenatal care, or neonatal care. Within the current studies, it is not possible to determine which of these is most likely. Evidence from the VON also suggests that the mortality rate for infants with SIP is lower than that of infants with NEC^4^, whereas within our cohort, infants diagnosed with SIP at laparotomy had a higher risk of 28-day mortality than infants with NEC. This difference appears to be being driven by the fact that infants with laparotomy confirmed NEC in our cohort have a significantly lower mortality rate than those in the VON cohort, whereas infants with SIP have a similar mortality rate. This could reflect three things. Firstly, that infants with NEC are more prone to late mortality, whilst infants with SIP experience early mortality, and therefore, when a one-year analysis of this cohort is undertaken, a similar picture to the VON studies may be demonstrated. Secondly, it may again reflect the potentially different populations recruited to the study, based upon differing thresholds for PPD usage, laparotomy, and palliation. Thirdly, the mortality rate amongst infants with laparotomy confirmed NEC may genuinely be lower in the UK than in the US, whilst rates of mortality from laparotomy confirmed SIP are similar. Again, this could be driven by differences in antenatal, neonatal or surgical care, and it is not currently possible to determine which.

Members of the Parent Advisory Group established by the National Perinatal Epidemiology Unit frequently report that one of the most distressing aspects of having an infant who requires neonatal surgery is that dependent on the clinician they speak to, they receive differing advice relating to their babies management and prognosis. The apparent differences in outcomes between this study, and those of the VON highlight how important it is to have population specific information on which to base such counselling. This Nationwide cohort study can help address this issue through providing key information that can be used universally by clinicians across the United Kingdom to counsel parents of infants with NEC on the likely pattern of their infant’s early disease course. Specific information from this study that can be used for counselling includes the fact that approximately 1 in four infants diagnosed with NEC in the UK and Ireland will require surgical intervention, that if an infant requires surgical intervention, they are most likely to undergo resection of a segment of bowel and formation of a stoma, and that post-operatively, approximately 80% of infants will survive to 28 days post-intervention. They can also be provided with information to illustrate the difficult nature of the surgical intervention, including the fact that approximately one quarter of infants undergoing a laparotomy will develop a complication as a result of it. As well as counselling parents, this information can be used for benchmarking of outcomes. Within the UK, there is a drive by the Royal College of Surgeons for surgeon specific outcomes to be used for revalidation purposes. For neonatal surgeons, early outcomes for infants with NEC are proposed as one measure that will be used. The robust national data collected in this study provides a national benchmark against which surgeons can compare their own performance, and also provides information on factors that should be taken into account when assessing a surgeons case mix for the purposes of comparison against such a national benchmark. Despite the identification of associations between key pre-operative and operative factors that have been highlighted in this study, there are still many more that are left unidentified. Investigating these further with large-scale, international cohort studies that combine the depth of the BAPS-CASS studies, with the scale of the Vermont Oxford Network studies will be an essential next step in improving outcomes for infants with surgical NEC.

## Additional Information

**How to cite this article**: Allin, B. *et al*. A UK wide cohort study describing management and outcomes for infants with surgical Necrotising Enterocolitis. *Sci. Rep.* **7**, 41149; doi: 10.1038/srep41149 (2017).

**Publisher's note:** Springer Nature remains neutral with regard to jurisdictional claims in published maps and institutional affiliations.

## Supplementary Material

Supplementary Information

## Figures and Tables

**Table 1:** Characteristics of included infants.

		Number of infants (%)
Characteristics		Overall
Sex	Male	145 (61%)
	Female	91 (39%)
Ethnicity*	White British	125 (57%)
	Other	93 (43%)
Birth-Weight (g)	Median (IQR)	910 g (730g–1375g)
Gestational age at birth** (Completed weeks)	≥37	17 (7%)
	32 < 37	26 (11%)
	28 < 32	54 (23%)
	26 < 28	54 (23%)
	<26	84 (36%)

^*^Missing data for eight infants.

^**^Missing data for one infant.

**Table 2:** Surgical interventions undertaken.

Management	Number of infants		
	Cohort as a whole	NEC confirmed at laparotomy#	SIP confirmed at laparotomy#
Total	236*	189*	32
No intervention	6 (3%)	N/A	N/A
Primary peritoneal drainage alone	6 (3%)	N/A	N/A
Resection and primary anastomosis	40 (17%)	31 (17%)	9 (28%)
Resection and stoma formation	121 (52%)	105 (56%)	15 (47%)
Stoma formation without resection	38 (16%)	30 (16%)	7 (22%)
Clip and drop with resection	10 (4%)	9 (5%)	1 (3%)
Open and close laparotomy	10 (4%)	10 (5%)	0 (0%)
Negative initial laparotomy	2 (1%)	2 (1%)	0 (0%)

^*^Missing information on procedure performed for three infants who underwent a laparotomy. Two were confirmed to have NEC, and in one the diagnosis was unclear.

^#^Missing information on final diagnosis in three infants who underwent laparotomy.

NEC – Necrotising Enterocolitis. SIP – Spontaneous Intestinal Perforation.

**Table 3:** Mortality, discharge and use of TPN according to definitive diagnosis.

	Entire cohort	Infants with laparotomy confirmed NEC	Infants with laparotomy confirmed SIP	Infants not undergoing laparotomy
Total number of infants	236*	189 (80%)	32 (14%)	12 (5%)
Number of infants who died prior to 28 days	43 (18%)	29 (15%)	7 (22%)	7 (58%)
Number of infants who were alive and parenteral nutrition free at 28 days	67 (35%)	55 (34%)	11 (44%)	5 (100%)
Number of infants discharged home prior to 28 days	8 (3%)	7 (4%)	1 (3%)	0 (0%)
Number of infants undergoing laparotomy who developed a Clavien-Dindo grade two or above complication prior to 28 days*	60 (27%)	48 (25%)	11 (34%)	N/A

^*^Missing information on final diagnosis in three infants who underwent a laparotomy, one of whom developed a post-operative complication.

NEC – Necrotising Enterocolitis. SIP – Spontaneous Intestinal Perforation.

**Table 4:** Pre-operative characteristics meeting criteria for inclusion in the multivariable regression analysis model, and their association with 28-day mortality.

Pre-operative Characteristic		Died n (%)	Alive n (%)	OR (95% CI)	Adjusted OR (95% CI)*
Gestational age (per completed week)		0.98 (0.90–1.1)			0.94 (0.86–1.03)
Abdominal wall erythema or discolouration at presentation	Yes	20 (47%)	52 (27%)	2.34 (1.11–4.86)	2.51 (1.23–5.1)
	No	23 (53%)	140 (73%)		
Non-cardiac congenital anomaly	Yes	10 (24%)	16 (8%)	3.44 (1.27–8.87)	5.17 (1.9–14.1)
	No	32 (76%)	176 (92%)		
PDA ligation performed	Yes	1 (2%)	17 (9%)	0.25 (0.006–1.70)	0.74 (0.02–1.36)
	No	41 (98%)	175 (91%)		

^*^As complete case analysis was used for development of the final model, data from two infants (1%) were not used in its development.

PDA – Patent Ductus Arteriosus.

**Table 5:** Operative characteristics included in the multi-variable model, and their association with 28-day mortality.

Operative Characteristic		Died n(%)	Alive n(%)	Odds Ratio (95% CI)	Adjusted Odds Ratio (95% CI)*
Ileo-caecal valve resected	Yes	2 (6%)	38 (21%)	0.2 (0.03–0.96, p = 0.03)	0.2 (0.04–1.4)
	No	34 (94%)	147 (79%)		
Definitive diagnosis of SIP at laparotomy	Yes	7 (19%)	25 (14%)	1.5(0.5–4.1, p = 0.35)	3.1 (1.05–9.3)
	No	29 (81%)	160 (86%)		
Operation performed	Resection and primary anastomosis	2 (6%)	38 (21%)	1 (reference)	1 (reference)
	Resection and stoma formation	12 (33%)	108 (59%)	2.1 (0.45–9.9, p = 0.34)	2.4 (0.5–12.1)
	Stoma formation, no resection	7 (19%)	30 (16%)	4.4 (0.86–22.9, p = 0.08)	4.5 (0.8–26)
	Clip and drop with resection	5 (14%)	5 (3%)	19 (2.9–125.3, p = 0.002)	30 (3.9–237)
	Open and close laparotomy	10 (28%)	0 (0%)	Not calculable	Not calculable
	Negative initial laparotomy	0 (0%)	2 (11%)	Not calculable	Not calculable

^*^Complete case analysis was used for development of the final model, and therefore data from 17 of the 224 infants who underwent a laparotomy (7.5%) were not used in its development.

SIP – Spontaneous Intestinal Perforation.
