# Corrigendum: A UK wide cohort study describing management and outcomes for infants with surgical Necrotising Enterocolitis

**DOI:** 10.1038/srep46876

**Published:** 2017-07-17

**Authors:** Benjamin Allin, Anna-May Long, Amit Gupta, Marian Knight, Kokila Lakhoo, British Association of Paediatric Surgeons Congenital Anomalies Surveillance System Necrotising Enterocolitis Collaboration, Marcin Kazmierski, Simon Kenny, Joana Lopes, Eleri Cusick, Gilian Parsons, Amanda McCabe, Manasvi Upadhyaya, Gregor Walker, Paulo De Coppi, Sania Besarovic, Hemanshoo Thakkar, Lucinda Tullie, Jonathan Sutcliffe, Bala Eradi, Andrew Ross, Nomsa Maphango, Sandeep Motiwale, Adnan Salloum, Caroline Pardy, Ramy Waly, Paul Charlesworth, Ross Craigie, Anupam Lall, Richard Lindley, Navroop Johal, Ike Njere, Alan Mortell, Bip Nandi, Abigail Jones, Dina Fouad, Yew-Wei tan, Dorothy Kufeji, Joanna Stanwell, Bhanu Lakshminarayanan, David Burge, Charlotte Wetherill, Anindya Niyogi, Chris Parsons, Miriam Doyle, Alex Turner, Ian Yardley, Ram Shrestha, Dhanya Mullassery, Saravankumar Paramalingham, Simone Ragazzi

Scientific Reports 7: Article number: 41149; 10.1038/srep41149 published online: 01 27 2017; updated: 07 17 2017.

The Acknowledgements section in this Article is incomplete.

“This article is submitted as an observational study in the category of original articles. A small sub-set of information from this article has been presented at the annual congress of the British Association of Paediatric Surgeons”.

should read:

“This article is submitted as an observational study in the category of original articles. A small sub-set of information from this article has been presented at the annual congress of the British Association of Paediatric Surgeons. Marian Knight is funded by a National Institute for Health Research (NIHR) Professorship. Benjamin Allin is funded by a National Institute for Health Research (NIHR) Doctoral Research Fellowship. The views expressed are those of the author(s) and not necessarily those of the NHS, the NIHR or the Department of Health. The NIHR had no role in design and conduct of the study; collection, management, analysis, and interpretation of the data; and preparation, review, or approval of the manuscript; and decision to submit the manuscript for publication”.

