# Supplementary material for: A UK wide cohort study describing management and outcomes for infants with surgical Necrotising Enterocolitis
Source: Sci Rep. 2017 Jan 27;7:41149. doi: 10.1038/srep41149 (PMC5269581; doi:10.1038/srep41149)
Supplement: Supplementary Information [file srep41149-s1.doc]

**Supplementary information for**

**A UK wide cohort study describing management and outcomes for infants with surgical Necrotising Enterocolitis**

Benjamin Allin^1,2^, Anna-May Long^1,2^, Amit Gupta^3^#, Marian Knight^1^#, Kokila Lakhoo^2^#, British Association of Paediatric Surgeons Congenital Anomalies Surveillance System Necrotising Enterocolitis Collaboration*

#Joint senior authors

* The members of the BAPS-CASS group are collaborators on this study, and their names can be identified in the collaborators section

Benjamin Allin MBBS BSc MRCS, NIHR Doctoral Research Fellow, Honorary paediatric surgical registrar

Anna-May Long MBBS, Clinical Research Fellow, anna-may.long@gtc.ox.ac.uk

Amit Gupta, Consultant Neonatologist, amit.gupta@ouh.nhs.uk

Marian Knight MA MBChB MPH DPhil FFPH FRCPE, NIHR Professor of Maternal and Child Population Health, marian.knight@npeu.ox.ac.uk

Kokila Lakhoo, MBCHB, PhD, FRCS(ENG+EDIN), FCS(SA), MRCPCH(UK), Consultant Paediatric Surgeon, kokila.lakhoo@ouh.nhs.uk

^1^National Perinatal Epidemiology Unit, Old Road Campus, Headington, Oxford, OX37LF

^2^Department of Paediatric Surgery, Oxford Children’s Hospital, Headley Way, Oxford, OX39DU

^3^Neonatal Intensive Care Unit, Oxford Children’s Hospital, Headley Way, Oxford, OX39DU

Correspondence and requests for reprints should be addressed to Benjamin Allin. Email: [benjamin.allin@npeu.ox.ac.uk](mailto:benjamin.allin@npeu.ox.ac.uk), Post: National Perinatal Epidemiology Unit, Old Road Campus, University of Oxford, Headington, Oxford, OX3 7LF, Tel: 01865617824

**Supplementary Table 1. Association of preoperative variables with twenty-eight day mortality**

| **Characteristic** | | **Died**  n (%)* | **Alive**  **n (%)*** | **OR (95% CI)** |
| --- | --- | --- | --- | --- |
| **White British** | Yes | 20 (50%) | 105 (59%) | 0.70 (0.33-1.47) |
|  | No | 20 (50%) | 73 (41%) |  |
| **Male** | Yes | 27 (63%) | 118 (61%) | 1.06 (0.51-2.25) |
|  | No | 16 (37%) | 74 (39%) |  |
| **Reversed End Diastolic Flow** | Yes | 3 (21%) | 8 (16%) | 1.43 (0.21-7.34) |
|  | No | 11 (79%) | 42 (84%) |  |
| **Absent End Diastolic Flow** | Yes | 3 (21%) | 17 (34%) | 0.53 (0.08-2.41) |
|  | No | 11 (79%) | 33 (66%) |  |
| **Antenatal corticosteroids** | Yes | 26 (72%) | 121 (72%) | 1.01 (0.43-2.53) |
|  | No | 10 (28%) | 47 (28%) |  |
| **Mode of delivery** | Caesarean | 16 (37%) | 82 (43%) | 0.8 (0.4-1.6) |
|  | Vaginal | 27 (63%) | 110 (57%) |  |
| **Multip birth** | Yes | 12 (28%) | 41 (21%) | 1.4 (0.6-3.2) |
|  | No | 31 (72%) | 151 79%) |  |
| **APGAR<5 at 10 minutes** | Yes | 4 (12%) | 15 (9%) | 1.3 (0.3-4.6) |
|  | No | 30 (88%) | 149 (91%) |  |
| **Characteristic** | | **Died**  n (%)* | **Alive**  **n (%)*** | **OR (95% CI)** |
| **Gestational age (per completed week)#** | | 0.98 (0.90-1.1) | | |
| **Birth-weight (per 50g change)#** | | 0.99 (0.96-1.02) | | |
| **Pre-operative Evidence of perforation** | Yes | 22 (51%) | 90 (47%) | 1.19 (0.58-2.43) |
|  | No | 21 (49%) | 102 (53%) |  |
| **Abdominal wall erythema or discolouration at presentation** | Yes | 20 (47%) | 52 (27%) | 2.34 (1.11-4.86) |
|  | No | 23 (53%) | 140 (73%) |  |
| **Inotropes at presentation** | Yes | 14 (33%) | 41 (21%) | 1.83 (0.81-3.98) |
|  | No | 28 (67%) | 150 (79%) |  |
| **Ventilated at presentation** | Yes | 33 (77%) | 113 (59%) | 2.31 (1.03-5.54) |
|  | No | 10 (23%) | 79 (41%) |  |
| **Antacid use at presentation** | Yes | 4 (9%) | 15 (8%) | 1.18 (0.27-3.99) |
|  | No | 39 (91%) | 173 (92%) |  |
| **Non-PDA Cardiac Surgery** | Yes | 0 (0%) | 10 (5%) | 0 (0-1.66) |
|  | No | 43 (100%) | 182 (95%) |  |
| **Non-cardiac congenital anomaly** | Yes | 10 (24%) | 16 (8%) | 3.44 (1.27-8.87) |
|  | No | 32 (76%) | 176 (92%) |  |
| **Transferred in to surgical centre** | Yes | 29 (67%) | 150 (78%) | 0.58 (0.27-1.30) |
|  | No | 14 (33%) | 42 (22%) |  |
| **Characteristic** | | **Died**  n (%)* | **Alive**  **n (%)*** | **OR (95% CI)** |
| **PDA ligation performed** | Yes | 1 (2%) | 17 (9%) | 0.25 (0.006-1.70) |
|  | No | 41 (98%) | 175 (91%) |  |
| **Indomethacin for PDA Closure** | Yes | 9 (22%) | 41 (23%) | 0.95 (0.37-2.26) |
|  | No | 32 (88%) | 139 (77%) |  |
| **Umbilical Catheter ever used** | Yes | 22 (71%) | 92 (66%) | 1.28 (0.51-3.40) |
|  | No | 9 (29%) | 48 (34%) |  |
| **Ever enterally fed prior to diagnosis** | Yes | 37 (86%) | 162 (84%) | 1.14 (0.43-3.60) |
|  | No | 6 (14%) | 30 (16%) |  |
| **Formula milk at diagnosis** | Yes | 10 (23%) | 41 (21%) | 1.11 (0.45-2.56) |
|  | No | 33 (77%) | 151 (79%) |  |
| **Blood Transfusion <2 weeks prior to diagnosis** | Yes | 14 (35%) | 57 (32%) | 1.16 (0.52-2.51) |
|  | No | 26 (65%) | 123 (68%) |  |
| **Pre-operative drain inserted** | **Yes** | 12 (6%) | 3 (8%) | 1.3 (0.2-5.2, p=0.7) |
|  | **No** | 173 (94%) | 33 (92%) |  |
| **ICV resected** | **Yes** | 38 (21%) | 2 (6%) | 0.2 (0.03-0.96, p=0.03) |
|  | **No** | 147 (79%) | 34 (94%) |  |
| **Definitive diagnosis of SIP at laparotomy** | **Yes** | 25 (14%) | 7 (19%) | 1.5(0.5-4.1, p=0.35) |
|  | **No** | 160 (86%) | 29 (81%) |  |
| **Characteristic** | | **Died**  n (%)* | **Alive**  **n (%)*** | **OR (95% CI)** |
| **Operation performed** | **Resection and primary anastomosis** | 38 (21%) | 2 (6%) | 1 (reference) |
|  | **Resection and stoma formation** | 108 (59%) | 12 (33%) | 2.1 (0.45-9.9, p=0.34) |
|  | **Stoma formation, no resection** | 30 (16%) | 7 (19%) | 4.4 (0.86-22.9, p=0.08) |
|  | **Clip and drop with resection** | 5 (3%) | 5 (14%) | 19 (2.9-125.3, p=0.002) |
|  | **Open and close laparotomy** | 0 (0%) | 10 (28%) | Not calculable |
|  | **Negative initial laparotomy** | 2 (11%) | 0 (0%) | Not calculable |

*Percentages based on number with information for that variable
